# 4,4′-Bipyrid­yl–4,4′-(hy­droxy­methyl­ene)dibenzoic acid (1/1)

**DOI:** 10.1107/S1600536812011956

**Published:** 2012-03-24

**Authors:** Lan Qin, Lan-Ping Xu, Lei Han

**Affiliations:** a Faculty of Materials Science & Chemical Engineering, Ningbo University, Ningbo, Zhejiang 315211, People’s Republic of China

## Abstract

In the title 1:1 co-crystal, C_10_H_8_N_2_·C_15_H_12_O_5_, strong inter­molecular O—H⋯N hydrogen bonds link alternating mol­ecules of 4,4′-(hy­droxy­methyl­ene)dibenzoic acid and 4,4′-bipyridyl into zigzag chains in [501]. The crystal packing also exhibits π–π inter­actions between the 4,4′-bipyridyl rings of neighbouring chains [centroid–centroid distance = 3.608 (3) Å] and weak C—H⋯O hydrogen bonds.

## Related literature

For background to supra­molecular crystal engineering, see: Simon & Bassoul (2000 ▶). For aromatic carb­oxy­lic acids as supra­molecular synthons, see: Desiraju (1995 ▶). For studies of bent arenedicarboxyl­ate ligands, see: Koichi *et al.* (2011 ▶); Xu *et al.* (2011 ▶).
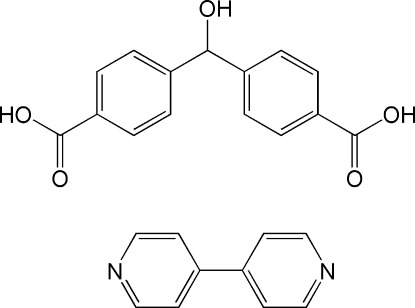


## Experimental

### 

#### Crystal data

C_10_H_8_N_2_·C_15_H_12_O_5_
*M* *_r_* = 428.43
Monoclinic, 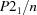

*a* = 8.0528 (16) Å
*b* = 11.683 (2) Å
*c* = 21.922 (4) Å
β = 96.66 (3)°
*V* = 2048.6 (7) Å^3^
*Z* = 4
Mo *K*α radiation
μ = 0.10 mm^−1^
*T* = 298 K
0.31 × 0.14 × 0.12 mm

#### Data collection

Rigaku R-AXIS RAPID diffractometer
Absorption correction: multi-scan (*ABSCOR*; Higashi, 1995 ▶) *T*_min_ = 0.984, *T*_max_ = 0.988
16322 measured reflections
3800 independent reflections
1903 reflections with *I* > 2σ(*I*)
*R*_int_ = 0.089

#### Refinement

*R*[*F* ^2^ > 2σ(*F* ^2^)] = 0.077
*wR*(*F* ^2^) = 0.210
*S* = 0.99
3800 reflections
298 parameters
H atoms treated by a mixture of independent and constrained refinement
Δρ_max_ = 0.68 e Å^−3^
Δρ_min_ = −0.28 e Å^−3^

### 

Data collection: *RAPID-AUTO* (Rigaku, 1998 ▶); cell refinement: *RAPID-AUTO*; data reduction: *CrystalStructure* (Rigaku/MSC, 2004 ▶); program(s) used to solve structure: *SHELXS97* (Sheldrick, 2008 ▶); program(s) used to refine structure: *SHELXL97* (Sheldrick, 2008 ▶); molecular graphics: *SHELXTL* (Sheldrick, 2008 ▶); software used to prepare material for publication: *SHELXL97*.

## Supplementary Material

Crystal structure: contains datablock(s) I, global. DOI: 10.1107/S1600536812011956/cv5265sup1.cif

Structure factors: contains datablock(s) I. DOI: 10.1107/S1600536812011956/cv5265Isup2.hkl

Supplementary material file. DOI: 10.1107/S1600536812011956/cv5265Isup3.cml

Additional supplementary materials:  crystallographic information; 3D view; checkCIF report

## Figures and Tables

**Table 1:** Hydrogen-bond geometry (Å, °)

*D*—H⋯*A*	*D*—H	H⋯*A*	*D*⋯*A*	*D*—H⋯*A*
O2—H2⋯N2^i^	0.84 (5)	1.82 (5)	2.660 (5)	173 (5)
O4—H1⋯N1^ii^	0.87 (6)	1.76 (6)	2.605 (5)	166 (5)
C19—H19*A*⋯O3^iii^	0.93	2.40	3.321 (5)	171
C17—H17*A*⋯O1^iv^	0.93	2.57	3.212 (5)	126
C8—H8*A*⋯O3^iv^	0.98	2.51	3.376 (6)	148

Symmetry codes: (i) 
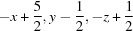
; (ii) 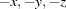; (iii) 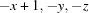; (iv) 
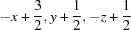
.

**Fig. 1.**
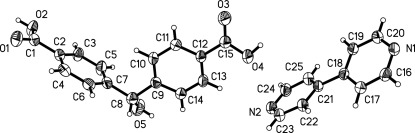
A content of asymmetric unit of the title compound showing the atomic numbering and 30% probability displacement ellipsoids.

**Fig. 2.**
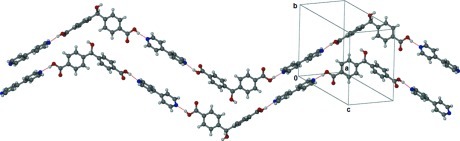
A portion of the crystal packing showing O—H···N hydrogen bonds as dashed lines.

## supplementary crystallographic information

### Comment

Supramolecular crystal engineering has attracted growing interest over the past few decades because of their importance in biological system and molecular recognition (Simon *et al.*, 2000). Aromatic carboxylic acid is one of the most important supramolecular synthons to construct novel organic networks by hydrogen bonds and π–π interactions (Desiraju, 1995). Recently, interest has been devoted to the assembly of extended solids from the long and bent arenedicarboxylate ligands (Koichi *et al.*, 2011; Xu *et al.*, 2011). We have employed 4,4'-(hydroxymethylene)dibenzoic acid as an excellent candidate for the construction of targeted supramolecular structures. A new organic cocrystal compound, C_15_H_12_O_5_.C_10_H_8_N_2_, has been synthesized by reacting 4,4'-(hydroxymethylene)dibenzoic acid and 4,4'-bipyridyl under hydrothermal conditions, and its crystal structure is reported here.

The asymmetric unit of the title compound consists of one 4,4'-(hydroxymethylene)dibenzoic acid and one 4,4'-bipyridyl molecule (Figure 1). The dihedral angle formed by two pyridine rings in 4,4'-bipyridyl is 24.74 (1)° , and the dihedral angle between the two benzene rings in bent 4,4'-(hydroxymethylene)dibenzoic acid ligand is 85.95 (3)° . In the1:1 cocrystal, strong intermolecular O—H···N hydrogen bonds link the alternating molecules of 4,4'-(hydroxymethylene)dibenzoic acid and 4,4'-bipyridyl into zigzag chains in [501], as shown in Figure 2. Furthermore, the crystal packing exhibits also π–π interactions between the rings of 4,4'-bipyridyl from the neighbouring chains [centroid–centroid distance of 3.608 (3) Å] and weak C—H···O hydrogen bonds.

### Experimental

A mixture of 4,4'-(hydroxymethylene)dibenzoic acid (26.4 mg, 0.1 mmol) and 4,4'-bipyridyl (15.1 mg, 0.1 mmol) in H_2_O (8 ml) was sealed in a 25 ml Teflon-lined stainless steel reactor and heated at 447 K for 3 d. Colorless single crystals of the title compound was obtained after cooling the solution to room temperature. Block-shaped crystals were collected and washed with distilled water. The yield was approximately 70% based on 4,4'-(hydroxymethylene)dibenzoic acid.

### Refinement

H atoms attached to O2 and O4 were located in difference maps and refined isotropically. C-bound H atoms were positioned geometrically and allowed to ride on their respective parent atoms at distances of C—H = 0.93 Å, with *U*_iso_(H) = 1.2*U*eq(C).

### Crystal data

**Table:**

C_10_H_8_N_2_·C_15_H_12_O_5_	*F*(000) = 896
*M**_r_* = 428.43	*D*_x_ = 1.389 Mg m^−^^3^
Monoclinic, *P*2_1_/*n*	Mo *K*α radiation, λ = 0.71073 Å
Hall symbol: -P 2yn	Cell parameters from 2687 reflections
*a* = 8.0528 (16) Å	θ = 3.1–25.5°
*b* = 11.683 (2) Å	µ = 0.10 mm^−^^1^
*c* = 21.922 (4) Å	*T* = 298 K
β = 96.66 (3)°	Block, colourless
*V* = 2048.6 (7) Å^3^	0.31 × 0.14 × 0.12 mm
*Z* = 4	

### Data collection

**Table:**

Rigaku R-AXIS RAPID diffractometer	3800 independent reflections
Radiation source: fine-focus sealed tube	1903 reflections with *I* > 2σ(*I*)
Graphite monochromator	*R*_int_ = 0.089
Detector resolution: 0 pixels mm^-1^	θ_max_ = 25.5°, θ_min_ = 3.1°
ω scans	*h* = −9→9
Absorption correction: multi-scan (*ABSCOR*; Higashi, 1995)	*k* = −14→14
*T*_min_ = 0.984, *T*_max_ = 0.988	*l* = −26→24
16322 measured reflections	

### Refinement

**Table:**

Refinement on *F*^2^	Secondary atom site location: difference Fourier map
Least-squares matrix: full	Hydrogen site location: inferred from neighbouring sites
*R*[*F*^2^ > 2σ(*F*^2^)] = 0.077	H atoms treated by a mixture of independent and constrained refinement
*wR*(*F*^2^) = 0.210	*w* = 1/[σ^2^(*F*_o_^2^) + (0.0824*P*)^2^ + 1.4919*P*] where *P* = (*F*_o_^2^ + 2*F*_c_^2^)/3
*S* = 0.99	(Δ/σ)_max_ < 0.001
3800 reflections	Δρ_max_ = 0.68 e Å^−^^3^
298 parameters	Δρ_min_ = −0.28 e Å^−^^3^
0 restraints	Extinction correction: *SHELXL97* (Sheldrick, 2008), Fc^*^=kFc[1+0.001xFc^2^λ^3^/sin(2θ)]^-1/4^
Primary atom site location: structure-invariant direct methods	Extinction coefficient: 0.0067 (18)

### Special details

**Table:**

Geometry. All esds (except the esd in the dihedral angle between two l.s. planes) are estimated using the full covariance matrix. The cell esds are taken into account individually in the estimation of esds in distances, angles and torsion angles; correlations between esds in cell parameters are only used when they are defined by crystal symmetry. An approximate (isotropic) treatment of cell esds is used for estimating esds involving l.s. planes.
Refinement. Refinement of F^2^ against ALL reflections. The weighted R-factor wR and goodness of fit S are based on F^2^, conventional R-factors R are based on F, with F set to zero for negative F^2^. The threshold expression of F^2^ > 2sigma(F^2^) is used only for calculating R-factors(gt) etc. and is not relevant to the choice of reflections for refinement. R-factors based on F^2^ are statistically about twice as large as those based on F, and R- factors based on ALL data will be even larger.

### Fractional atomic coordinates and isotropic or equivalent isotropic displacement parameters (Å^2^)

**Table:**

	*x*	*y*	*z*	*U*_iso_*/*U*_eq_	
O1	1.7007 (4)	−0.0589 (3)	0.44854 (15)	0.0828 (10)	
O2	1.8293 (4)	−0.0048 (3)	0.36999 (15)	0.0844 (11)	
O3	0.6425 (4)	−0.0831 (3)	0.12982 (16)	0.0831 (10)	
O4	0.5017 (4)	0.0793 (3)	0.11948 (17)	0.0863 (12)	
O5	1.1910 (4)	0.3660 (3)	0.2676 (2)	0.1155 (15)	
H5A	1.1098	0.4045	0.2542	0.173*	
N1	−0.2656 (4)	0.0472 (3)	−0.06295 (16)	0.0628 (10)	
N2	0.4192 (4)	0.3635 (3)	0.08219 (19)	0.0694 (10)	
C1	1.7018 (5)	−0.0067 (4)	0.4015 (2)	0.0610 (11)	
C2	1.5595 (5)	0.0650 (4)	0.37421 (18)	0.0557 (11)	
C3	1.5610 (5)	0.1201 (4)	0.31932 (19)	0.0686 (13)	
H3A	1.6546	0.1139	0.2984	0.082*	
C4	1.4188 (5)	0.0754 (4)	0.40432 (19)	0.0633 (12)	
H4A	1.4142	0.0385	0.4417	0.076*	
C5	1.4258 (5)	0.1850 (5)	0.2941 (2)	0.0780 (15)	
H5B	1.4302	0.2220	0.2568	0.094*	
C6	1.2844 (5)	0.1406 (4)	0.3790 (2)	0.0668 (12)	
H6A	1.1908	0.1475	0.3999	0.080*	
C7	1.2865 (5)	0.1952 (4)	0.3238 (2)	0.0653 (12)	
C8	1.1347 (5)	0.2650 (4)	0.2987 (2)	0.0784 (15)	
H8A	1.0768	0.2904	0.3332	0.094*	
C9	1.0110 (4)	0.1983 (4)	0.25372 (18)	0.0586 (11)	
C10	1.0175 (5)	0.0821 (4)	0.24747 (19)	0.0660 (12)	
H10A	1.1054	0.0415	0.2686	0.079*	
C11	0.8944 (5)	0.0235 (4)	0.20995 (19)	0.0613 (11)	
H11A	0.9000	−0.0557	0.2064	0.074*	
C12	0.7646 (4)	0.0829 (4)	0.17821 (17)	0.0526 (10)	
C13	0.7589 (5)	0.1998 (4)	0.18338 (18)	0.0608 (11)	
H13A	0.6723	0.2404	0.1614	0.073*	
C14	0.8806 (5)	0.2575 (4)	0.22089 (19)	0.0618 (11)	
H14A	0.8752	0.3368	0.2242	0.074*	
C15	0.6312 (5)	0.0179 (4)	0.1404 (2)	0.0616 (11)	
C16	−0.2813 (5)	0.1129 (4)	−0.0146 (2)	0.0671 (12)	
H16A	−0.3849	0.1154	0.0003	0.081*	
C17	−0.1537 (5)	0.1778 (4)	0.01501 (19)	0.0594 (11)	
H17A	−0.1712	0.2220	0.0489	0.071*	
C18	0.0024 (4)	0.1760 (3)	−0.00674 (18)	0.0515 (10)	
C19	0.0182 (5)	0.1095 (3)	−0.05812 (18)	0.0578 (11)	
H19A	0.1191	0.1070	−0.0748	0.069*	
C20	−0.1179 (5)	0.0466 (4)	−0.0844 (2)	0.0641 (12)	
H20A	−0.1051	0.0020	−0.1187	0.077*	
C21	0.1463 (5)	0.2406 (3)	0.02397 (18)	0.0526 (10)	
C22	0.1556 (5)	0.2692 (4)	0.0859 (2)	0.0649 (12)	
H22A	0.0711	0.2478	0.1091	0.078*	
C23	0.2930 (5)	0.3303 (4)	0.1123 (2)	0.0725 (13)	
H23A	0.2973	0.3492	0.1536	0.087*	
C24	0.4097 (5)	0.3339 (4)	0.0235 (2)	0.0679 (13)	
H24A	0.4970	0.3555	0.0017	0.082*	
C25	0.2790 (5)	0.2731 (4)	−0.0073 (2)	0.0631 (12)	
H25A	0.2798	0.2542	−0.0484	0.076*	
H2	1.915 (6)	−0.042 (5)	0.385 (2)	0.100 (19)*	
H1	0.436 (7)	0.031 (5)	0.099 (2)	0.11 (2)*	

### Atomic displacement parameters (Å^2^)

**Table:**

	*U*^11^	*U*^22^	*U*^33^	*U*^12^	*U*^13^	*U*^23^
O1	0.084 (2)	0.091 (3)	0.071 (2)	0.0073 (18)	0.0029 (17)	0.0258 (19)
O2	0.061 (2)	0.109 (3)	0.082 (2)	0.023 (2)	0.0036 (18)	0.026 (2)
O3	0.0630 (19)	0.078 (3)	0.105 (3)	−0.0035 (17)	−0.0046 (17)	−0.008 (2)
O4	0.062 (2)	0.083 (3)	0.106 (3)	0.0024 (18)	−0.0254 (19)	−0.017 (2)
O5	0.089 (3)	0.090 (3)	0.156 (4)	−0.001 (2)	−0.033 (2)	0.012 (3)
N1	0.053 (2)	0.064 (2)	0.068 (2)	0.0002 (17)	−0.0067 (18)	−0.0012 (19)
N2	0.054 (2)	0.068 (3)	0.083 (3)	−0.0012 (18)	−0.007 (2)	−0.005 (2)
C1	0.057 (3)	0.068 (3)	0.057 (3)	−0.005 (2)	−0.002 (2)	−0.001 (2)
C2	0.051 (2)	0.064 (3)	0.051 (2)	−0.0029 (19)	0.0005 (19)	0.000 (2)
C3	0.053 (2)	0.095 (4)	0.058 (3)	0.014 (2)	0.009 (2)	0.012 (3)
C4	0.066 (3)	0.073 (3)	0.050 (2)	−0.013 (2)	0.007 (2)	−0.004 (2)
C5	0.064 (3)	0.109 (4)	0.060 (3)	0.015 (3)	0.002 (2)	0.015 (3)
C6	0.047 (2)	0.085 (3)	0.070 (3)	−0.001 (2)	0.010 (2)	−0.020 (3)
C7	0.052 (2)	0.079 (3)	0.063 (3)	0.002 (2)	−0.007 (2)	−0.015 (2)
C8	0.063 (3)	0.074 (4)	0.094 (4)	0.007 (2)	−0.011 (3)	−0.014 (3)
C9	0.045 (2)	0.071 (3)	0.059 (2)	0.002 (2)	0.0015 (19)	−0.012 (2)
C10	0.051 (2)	0.077 (3)	0.065 (3)	0.010 (2)	−0.011 (2)	−0.007 (2)
C11	0.058 (2)	0.059 (3)	0.064 (3)	0.005 (2)	0.000 (2)	−0.002 (2)
C12	0.044 (2)	0.064 (3)	0.049 (2)	−0.0003 (19)	0.0033 (17)	0.001 (2)
C13	0.048 (2)	0.074 (3)	0.058 (2)	0.006 (2)	−0.0040 (19)	0.000 (2)
C14	0.056 (2)	0.061 (3)	0.066 (3)	0.003 (2)	−0.001 (2)	−0.003 (2)
C15	0.055 (3)	0.064 (3)	0.065 (3)	0.001 (2)	0.001 (2)	−0.001 (2)
C16	0.047 (2)	0.068 (3)	0.086 (3)	0.001 (2)	0.005 (2)	−0.002 (3)
C17	0.054 (2)	0.061 (3)	0.062 (3)	0.003 (2)	0.001 (2)	−0.002 (2)
C18	0.048 (2)	0.051 (3)	0.053 (2)	0.0009 (18)	−0.0034 (18)	0.005 (2)
C19	0.053 (2)	0.059 (3)	0.059 (3)	0.003 (2)	−0.001 (2)	0.002 (2)
C20	0.063 (3)	0.066 (3)	0.060 (3)	0.003 (2)	−0.005 (2)	−0.003 (2)
C21	0.049 (2)	0.051 (3)	0.055 (2)	0.0005 (18)	−0.0044 (19)	0.0004 (19)
C22	0.056 (2)	0.065 (3)	0.071 (3)	0.000 (2)	−0.006 (2)	−0.004 (2)
C23	0.066 (3)	0.076 (3)	0.071 (3)	0.006 (2)	−0.012 (2)	−0.009 (3)
C24	0.052 (2)	0.070 (3)	0.080 (3)	−0.003 (2)	−0.002 (2)	0.004 (3)
C25	0.057 (2)	0.069 (3)	0.062 (3)	−0.003 (2)	0.000 (2)	0.003 (2)

### Geometric parameters (Å, º)

**Table:**

O1—C1	1.199 (5)	C9—C14	1.387 (5)
O2—C1	1.302 (5)	C10—C11	1.392 (5)
O2—H2	0.84 (5)	C10—H10A	0.9300
O3—C15	1.208 (5)	C11—C12	1.375 (5)
O4—C15	1.304 (5)	C11—H11A	0.9300
O4—H1	0.87 (6)	C12—C13	1.371 (6)
O5—C8	1.460 (6)	C12—C15	1.487 (6)
O5—H5A	0.8200	C13—C14	1.380 (5)
N1—C16	1.327 (5)	C13—H13A	0.9300
N1—C20	1.329 (5)	C14—H14A	0.9300
N2—C24	1.325 (6)	C16—C17	1.377 (5)
N2—C23	1.332 (6)	C16—H16A	0.9300
C1—C2	1.488 (6)	C17—C18	1.395 (5)
C2—C3	1.365 (5)	C17—H17A	0.9300
C2—C4	1.381 (6)	C18—C19	1.386 (5)
C3—C5	1.388 (6)	C18—C21	1.478 (5)
C3—H3A	0.9300	C19—C20	1.387 (5)
C4—C6	1.386 (6)	C19—H19A	0.9300
C4—H4A	0.9300	C20—H20A	0.9300
C5—C7	1.365 (6)	C21—C25	1.387 (6)
C5—H5B	0.9300	C21—C22	1.391 (5)
C6—C7	1.370 (6)	C22—C23	1.385 (6)
C6—H6A	0.9300	C22—H22A	0.9300
C7—C8	1.518 (6)	C23—H23A	0.9300
C8—C9	1.531 (6)	C24—C25	1.379 (6)
C8—H8A	0.9800	C24—H24A	0.9300
C9—C10	1.367 (6)	C25—H25A	0.9300
			
C1—O2—H2	116 (4)	C13—C12—C15	121.7 (4)
C15—O4—H1	104 (4)	C11—C12—C15	118.8 (4)
C8—O5—H5A	109.5	C12—C13—C14	120.5 (4)
C16—N1—C20	117.2 (4)	C12—C13—H13A	119.7
C24—N2—C23	116.3 (4)	C14—C13—H13A	119.7
O1—C1—O2	123.3 (4)	C13—C14—C9	120.5 (4)
O1—C1—C2	123.3 (4)	C13—C14—H14A	119.7
O2—C1—C2	113.3 (4)	C9—C14—H14A	119.7
C3—C2—C4	118.1 (4)	O3—C15—O4	123.0 (4)
C3—C2—C1	122.3 (4)	O3—C15—C12	122.7 (4)
C4—C2—C1	119.6 (4)	O4—C15—C12	114.3 (4)
C2—C3—C5	121.4 (4)	N1—C16—C17	124.0 (4)
C2—C3—H3A	119.3	N1—C16—H16A	118.0
C5—C3—H3A	119.3	C17—C16—H16A	118.0
C2—C4—C6	120.2 (4)	C16—C17—C18	118.9 (4)
C2—C4—H4A	119.9	C16—C17—H17A	120.5
C6—C4—H4A	119.9	C18—C17—H17A	120.5
C7—C5—C3	120.6 (4)	C19—C18—C17	117.2 (4)
C7—C5—H5B	119.7	C19—C18—C21	121.0 (4)
C3—C5—H5B	119.7	C17—C18—C21	121.8 (4)
C7—C6—C4	121.4 (4)	C18—C19—C20	119.5 (4)
C7—C6—H6A	119.3	C18—C19—H19A	120.3
C4—C6—H6A	119.3	C20—C19—H19A	120.3
C5—C7—C6	118.3 (4)	N1—C20—C19	123.1 (4)
C5—C7—C8	123.1 (5)	N1—C20—H20A	118.4
C6—C7—C8	118.5 (4)	C19—C20—H20A	118.4
O5—C8—C7	108.8 (4)	C25—C21—C22	117.3 (4)
O5—C8—C9	108.9 (4)	C25—C21—C18	121.6 (4)
C7—C8—C9	113.3 (4)	C22—C21—C18	121.2 (4)
O5—C8—H8A	108.6	C23—C22—C21	118.8 (4)
C7—C8—H8A	108.6	C23—C22—H22A	120.6
C9—C8—H8A	108.6	C21—C22—H22A	120.6
C10—C9—C14	118.6 (4)	N2—C23—C22	124.1 (5)
C10—C9—C8	122.7 (4)	N2—C23—H23A	117.9
C14—C9—C8	118.5 (4)	C22—C23—H23A	117.9
C9—C10—C11	121.0 (4)	N2—C24—C25	124.3 (4)
C9—C10—H10A	119.5	N2—C24—H24A	117.8
C11—C10—H10A	119.5	C25—C24—H24A	117.8
C12—C11—C10	119.9 (4)	C24—C25—C21	119.2 (4)
C12—C11—H11A	120.1	C24—C25—H25A	120.4
C10—C11—H11A	120.1	C21—C25—H25A	120.4
C13—C12—C11	119.5 (4)		

### Hydrogen-bond geometry (Å, º)

**Table:**

*D*—H···*A*	*D*—H	H···*A*	*D*···*A*	*D*—H···*A*
O2—H2···N2^i^	0.84 (5)	1.82 (5)	2.660 (5)	173 (5)
O4—H1···N1^ii^	0.87 (6)	1.76 (6)	2.605 (5)	166 (5)
C19—H19*A*···O3^iii^	0.93	2.40	3.321 (5)	171
C17—H17*A*···O1^iv^	0.93	2.57	3.212 (5)	126
C8—H8*A*···O3^iv^	0.98	2.51	3.376 (6)	148

Symmetry codes: (i) −*x*+5/2, *y*−1/2, −*z*+1/2; (ii) −*x*, −*y*, −*z*; (iii) −*x*+1, −*y*, −*z*; (iv) −*x*+3/2, *y*+1/2, −*z*+1/2.
